# The Matrix in Filling Teeth

**Published:** 1894-10

**Authors:** J. D. Patterson

**Affiliations:** Kansas City, Mo.


THE
AMERICAN JOURNAL
—OF—
DENTAL SCIENCE.

VOL. XXVIII. BALTIMORE, OCTOBER, 1894. No. 6.

ARTICLE I.

### THE MATRIX IN FILLING TEETH.

BY J. D. PATTERSON, D. D. S., KANSAS CITY, MO.

This instrument to me has become so indispensable that, should I be compelled to relinquish its use, I would feel very much at a loss how to proceed with any degree of approach to perfection, in very many cases. The dental engine or the rubber dam scarcely occupies a more important place, in my estimation, when we come to look at results secured with and without this instrument.

My first experience with the matrix was far from satisfactory. This was chiefly for the reason that I used a clumsy instrument, first introduced by Professor Louis Jack, and because I confined their use to gold fillings indiscriminately. I have, I think, been taught, after many years' experience, the proper province of the matrix, and my practice now has not changed for about eight years. Upon keeping an approximate record, I now find that about one-half the fillings I make are made with the assistance of the matrix. The average is about as follows:

For gold fillings the matrix is used in about 10 per cent of the cases. In plastic fillings—viz., amalgam and cement—the average will be about 75 per cent. This average has been greatly increased since I have found an admirable place for their use in approximal fillings of cement in the incisor teeth of young patients.

The objections to the matrix have been chiefly upon one point, and that is the impossibility of making the best adaptation of gold at the lateral walls in the coronal approximal cavity in which matrices are commonly used. The objection is one which has been well taken, and many failures at this point have been justly credited to the use of the matrix. Even this objection, however, can be overcome with the correct instrument and the proper form of gold. Those who decry the matrix, I have found, are those who use instruments unsuited to matrix filling, use a poorly shaped matrix, are wedded to cohesive foil, and have not used it at all in plastics. In gold-foil filling, he who uses exclusively cohesive foil has, I believe, little business with the matrix; at least, I know I have not, for the cavity must be extraordinarily easy of access if cohesive foil and the matrix have succeeded in making a perfect union of gold and tooth substance at the cervical and lateral walls.

My use of the matrix is quite exclusively confined to the plastic fillings, especially to cement. I consider it impossible to insert a good cement filling in any approximal cavity where the filling reaches the gum line or extends under it, without the matrix.

Some years since I made the assertion before this association that a cement filling, properly inserted, did not fail at the cervical border sooner than at any other point. The assertion was met with a storm of objections; the objectors, however, have, I believe, come to the belief that I stated the fact.

I am now prepared to go farther and say that when a cement filling is covered by the gum at the cervical border it is protected at that point and lasts longer there *than at any other point.* Experience and observation have *abundantly* proved this in my own practice.

Cement fillings, properly manipulated, are not dissolved out by any of the usual or unusual fluids or secretions found in the mouth, but by the mechanical attrition of mastication, the washing of the mouth fluids, the brushing of the tooth-brush, or picking with the tooth pick. *Ergo,* where cement fillings are protected from these forces, that part protected will be the least liable to waste. The perfection of the filling, however, at these points, which we formerly found so vulnerable, must be insured by the use of the suitable matrix, and can—so far as I know—be secured in no other way. Without the matrix, the filling at these vulnerable points is rarely perfect. It is pushed away while it is crystallizing, by the ligature, by the rubber dam, or by the gum, until anything but a perfect adaptation is present. With the properly adjusted and properly shaped matrix, which protects the cement thoroughly from any pressure of this kind, and insures perfect adaptation under pressure while the cement is hardening and after it is hard, we obtain at these vulnerable points as perfect a condition as at any other point of the filling.

Of the different kinds of matrices there are a multitude, and each one has its advantages and disadvantages. My preference for a simple matrix is that of Dr. Jack's improved forms. They are superior to others on account of the admirable anatomically formed concavities to contour the filling, and because they have the rigidity which I prefer in most cases—especially in gold. The use of the simple matrix has an objection, in that it is nearly always necessary to use a wedge for their retention and to force the metal close to the cervical border below the cavity. This wedging, when performed bunglingly, is a disagreeable and painful operation; but usually, when care and experience preside over the operation, there need be little pain produced, and no injury to the gum tissue. I will endeavor in my clinic to show how this may be accomplished.

What I have said of cement fillings applies quite as well to amalgam fillings in approximal cavities.

The simple matrix is only applicable in cavities where there are adjoining teeth. Where there are no adjoining teeth, or where the teeth are badly broken down, one or the other various forms of bane matrices upon the market, or one made at the bench to suit individual cases. Some of these I will endeavor to show in clinic.—*Western Dental Journal.*

